# Development of a Tri-Axial Cutting Force Sensor for the Milling Process

**DOI:** 10.3390/s16030405

**Published:** 2016-03-19

**Authors:** Yingxue Li, Yulong Zhao, Jiyou Fei, You Zhao, Xiuyuan Li, Yunxiang Gao

**Affiliations:** 1 The State Key Laboratory for Manufacturing Systems Engineering, Xi’an Jiaotong University, Xi’an 710049, China; yingxueli@stu.xjtu.edu.cn (Y.L.); zhaoyou319@stu.xjtu.edu.cn (Y.Z.); lixiuyuanxjtu@163.com (X.L.); gaoyunxiang627@stu.xjtu.edu.cn (Y.G.); 2 School of EMU Application and Maintenance Engineering, Dalian Jiaotong University, Dalian 116028, China; fjy@djtu.edu.cn

**Keywords:** three-component, fixed dynamometer, strain gauge, positional variation, milling force

## Abstract

This paper presents a three-component fixed dynamometer based on a strain gauge, which reduces output errors produced by the cutting force imposed on different milling positions of the workpiece. A reformative structure of tri-layer cross beams is proposed, sensitive areas were selected, and corresponding measuring circuits were arranged to decrease the inaccuracy brought about by positional variation. To simulate the situation with a milling cutter moving on the workpiece and validate the function of reducing the output errors when the milling position changes, both static calibration and dynamic milling tests were implemented on different parts of the workpiece. Static experiment results indicate that with standard loads imposed, the maximal deviation between the measured forces and the standard inputs is 4.87%. The results of the dynamic milling test illustrate that with identical machining parameters, the differences in output variation between the developed sensor and standard dynamometer are no larger than 6.61%. Both static and dynamic experimental results demonstrate that the developed dynamometer is suitable for measuring milling force imposed on different positions of the workpiece, which shows potential applicability in machining a monitoring system.

## 1. Introduction

Cutting force is a significant feature in milling procedure as it is closely related to process optimization [1], tool design [2,3], as well as tool condition monitoring [4], *etc*. A cutting force monitoring system could provide a fundamental reference for identification of milling conditions and also offer the basis for parameter modification or tool selection. As the first module to obtain force values, a tri-axial dynamometer for the milling process with appropriate accuracy is demanded.

Nowadays, the most prevailing commercial instrument to measure milling forces is piezoelectric dynamometer [5,6]. Though the dynamometer shows excellent performance, its complicated structure and co-working charge amplifier lead to an extraordinarily high price, which restricts its application fields mainly to academic research. In contrast with the expensive piezoelectric sensors, a strain gauge based dynamometer with much lower cost and simpler manufacturing process is a great prospect for industrial applications.

Based on the working conditions, multi-component force sensors with strain gauges are mainly divided into two categories. One is a rotating sensor that always revolves together with a spindle or tool [7,8,9], and the other is a fixed dynamometer, which is fastened on the milling table with a workpiece clamped on its upper plate. As additional attention has to be paid to issues caused by revolving structures, such as wireless data transmission [10], dynamic balance emendation, *etc.*, this paper focuses on the development of a fixed force sensor.

Though the fixed dynamometer has avoided most of the problems produced by revolving, the continual feeding and cutting on the workpiece poses a serious challenge to the output accuracy of the sensor. As is well known, the relationship between input and output of a multi-component force sensor is derived via calibration procedure, and the correlation is expressed in the form of a decoupling matrix. Therefore, the measuring accuracy largely depends on the precision of the decoupling matrix. Because the rotating sensor has an unmovable force bearing area connecting with the tool or spindle, only one decoupling matrix is easily obtained. When milling force is imposed on different parts of the workpiece during the machining process, in order to get accurate outputs in the fixed dynamometer, decoupling matrices need to be acquired for every point of the workpiece in theory. As the decoupling method is obviously infeasible, a fixed force sensor capable of decreasing the positional output errors is required. However, related studies on fixed dynamometers based on strain gauge usually do not concentrate on this issue. Three teams led by Korkut, Sağlam, and Murthy separately designed acquisition systems for milling forces, which selected the same sensor structure with octagonal rings as the spring element [11,12,13]. As a segment of data collection, the positional influence of the force sensor was not much involved. As collaborators with Sağlam, Yaldız *et al.* extended the sensor testing to four components [14]. Though the specific errors of desired signals under 1000 N inputs with an eccentricity of 50 mm were mentioned, the final decoupling result still focused on the central force solution. Ammar *et al.* designed a tri-axial force sensor for milling state identification [15], but their attention was not paid to the positional influence either. Furthermore, a decoupling matrix contains the proportional coefficient between the desired signal and cross interference produced, respectively, by measured force and cross coupling in the sensor output, and both of these elements varied with the movement of the force bearing points. To entirely evaluate the effect of the positional variation, the desired signal and cross interference both require careful measurement and comparison.

A three-component milling force sensor based on strain gauge is proposed in this paper in order to overcome the above issues. First, a method of reducing eccentric influence on sensor output was introduced via the model of a cross beam dynamometer; second, based on the method, a force sensor with three-layer cross beams was proposed, and its fabrication was described as well; finally, static calibration and dynamic milling tests were carried out to verify the performance of the developed sensor and validate the feasibility of positional influence reduction.

## 2. Materials and Methods

### 2.1. Design Methods

#### 2.1.1. Mechanical Model of Fixed Milling Sensors

**Figure 1:**
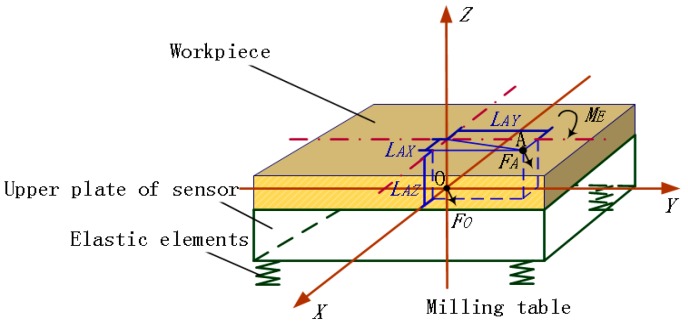
Diagram of eccentric force applied on a fixed dynamometer.

In a fixed dynamometer, compared with the elastic beams, the workpiece and the upper plate clamped together are considered as an integral rigid body. The mass center (Point O) of the suspended parts including top slab and elastic elements is taken as the origin point to build a global coordinate system as shown in Figure 1. When force *F_A_* is applied on an arbitrary point (Point A) of the workpiece, according to the principle of force translation on a rigid body [16], *F_A_* is expressed in Equation (1).

(1) $$\begin{array}{l} \vec{F_{A}}=\vec{F_{O}}+\vec{M_{E}}\\ \;=\;\vec{F_{OX}}\;+\;\vec{F_{OY}}\;+\;\vec{F_{OZ}}\;+\vec{\;M_{XY}}\;+\vec{\;M_{XZ}}\;+\;\;\vec{M_{YX}}\;+\vec{\;M_{YZ}}\;+\;\vec{M_{ZX}}\;+\;\vec{M_{ZY}} \end{array}$$

where *F_O_* denotes the force *F_A_* moving to Point O with the same magnitude and direction, and the additional moment is *M_E_*; *F_OX_*, *F_OY_*, and *F_OZ_* are the projections of *F_O_* on the *X*, *Y*, and *Z* axes. *M_XY_* and *M_YX_*, *M_XZ_* and *M_ZX_*, and *M_YZ_* and *M_ZY_* are moment components of *M_E_* separately in the *Z*, *Y*, and *X* directions; M_X*_, M_Y*_, and M_Z*_ are respectively generated by *X, Y*, and *Z* axial force components of *F_A_* (*i.e.*, *F_AX_*, *F_AY_*, and *F_AZ_*). The vector symbol will be ignored in the following context.

According to Equation (1), to keep small output variation under the forces with identical magnitude but different applied points (*i.e.*, the outputs brought by *F_O_* and *F_A_*), the measured signals generated from *M_E_* needs to be lessened and is best to be zero.

#### 2.1.2. Design of a Three-Layer Cross-Beam Force Sensor

Based on the mechanical model mentioned above, a three-layer cross-beam force sensor is developed, and a cross beam structure is selected as the basic elastic element of the dynamometer. With the combined action of sensor structure selection, sensitive area designation and measuring circuit building, the sensor is able to extract the signals produced by *F_O_* from the whole outputs generated by *F_A_*. Since the developed force sensor is perceived as a parallel connection of three cross beams, *M_E_* reducing method is understood more clearly with the model of a single cross beam structure.

##### *M_E_* Reduction Method in a Cross Beam Sensor

When the cross beam is adopted for a fixed sensor, its center platform takes the place of the upper plate where is used to clamp the workpiece. In the linear range of the sensor, the output signals generated by central force (*F_O_*) and additional moment (*M_E_*) are considered, and the final output is the superposition of all. The following analysis is derived under the hypothesis of simply supported beam, and all the stresses discussed below are along the axial directions of the elastic beams.

• Outputs under Central Force *F_O_*

**Figure 2:**
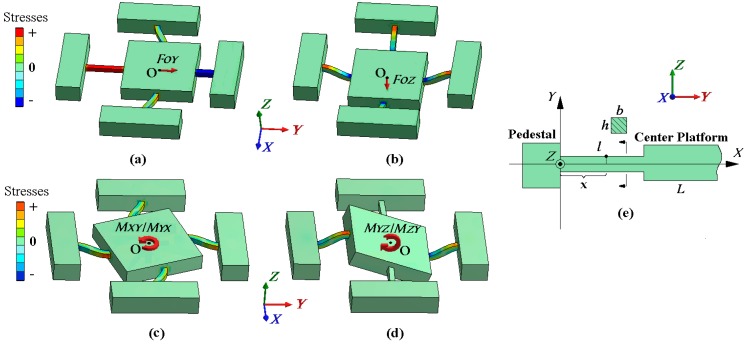
(**a**) Beam deformation under *F_OY_*; (**b**) beam deformation under *F_OZ_*; (**c**) beam deformation under *M_XY_* or *M_YX_*; (**d**) beam deformation under *M_YZ_* or *M_ZY_*; (**e**) local coordinate system for each elastic beam.

**Figure 3:**
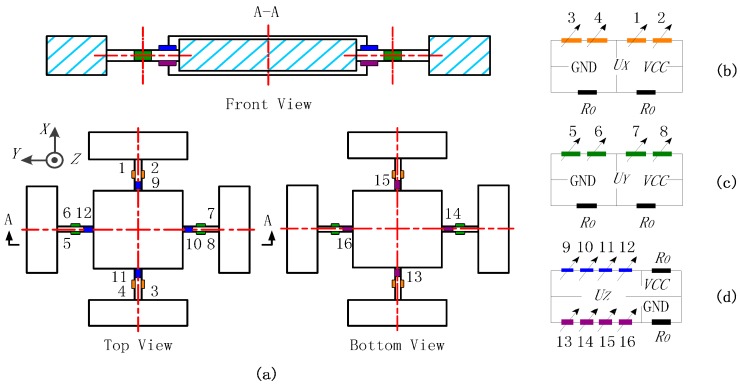
(**a**) Sensitive areas in a cross beam sensor; (**b**) Wheatstone half bridge circuit—Circuit X; (**c**) Wheatstone half bridge circuit—Circuit Y; (**d**) Wheatstone half bridge circuit—Circuit Z.

When a central force *F_O_* is applied on a cross beam sensor, the deformation diagrams under the components of *F_O_* are depicted in Figure 2a,b. The situation under *F_OX_* is omitted in view of the sensor symmetry. The stress concentrated regions to bound strain gauges (Areas 1–16) are all on the beam axes, as illustrated in Figure 3a. In the local coordinate system shown in Figure 2e, locations of Areas 1–8 are regarded as x = *l*/2; coordinate values of Areas 9–16 are considered as x ≈ *l*. The tension and compression on Areas 1–4 along the *X* axis are used to measure *F_OX_*, and the bending stresses on Areas 5–8 are idealized as zero, which is obtained under the abstract boundary condition that the rotation angles of their inside beam ends are constrained to zero. The stresses on Areas 1–8 under *F_OY_* are similar to the values analyzed under *F_OX_*. Vertical bending of elastic beams is utilized to measure *F_OZ_*, and the stresses of Areas 9–16 on neutral layers are not affected by *F_OX_* and *F_OY_*.

Three Wheatstone half bridge circuits (Circuit X, Y, and Z) are established for the cross beam sensor, as presented in Figure 3b–d. The fixed resistor used in the bridge arms is marked as *R_0_*, and the same resistance value of strain gauges is written as *R*. The output signals of the three measuring circuits under *F_O_* are given below:

(2) $$U_{X}(F_{O})=V_{CC}\left(\frac{2R+\sum_{i=3}^{4}\Delta r(i,F_{O})}{4R}-\frac{R_{0}}{2R_{0}}\right)=\frac{V_{CC}K}{4RE}(\sigma (3,F_{OX})+\sigma (4,F_{OX}))=\frac{V_{CC}Kl^{2}}{4REbh(b^{2}+l^{2})}F_{OX}$$

(3) $$U_{Y}(F_{O})=\frac{V_{CC}K}{4RE}(\sigma (7,F_{OY})+\sigma (8,F_{OY}))=\frac{V_{CC}Kl^{2}}{4REbh(b^{2}+l^{2})}F_{OY}$$

(4) $$U_{Z}(F_{O})=V_{CC}\left(\frac{4R+\sum_{i=13}^{16}\Delta r(i,F_{O})}{8R}-\frac{R_{0}}{2R_{0}}\right)=\frac{V_{CC}K}{8RE}\sum_{i=13}^{16}\sigma (i,F_{OZ})=-\frac{3V_{CC}Kl}{8REbh^{2}}F_{OZ}$$

where *U_X_(F_O_)*, *U_Y_(F_O_)*, and *U_Z_(F_O_)* stand for the output voltages from Circuit X, Y, and Z under *F_O_*. *Δr(i,)* is *F_O_* the resistance variation of a strain gauge and has two parameters in brackets; *i* represents the number of sensitive area, and *F_O_* denotes the force input. σ(i, F_*_) is the symbol of stress including the similar input parameters as explained in *Δr(i, F_O_)*; *V_CC_* is the supply voltage of measuring circuit; *K* is the working sensitivity coefficient of strain gauges; *b*, *h*, and *l* are the weight, height, and length of each beam; and *E* is the Young’s modulus of the material made for elastic beams.

- Outputs under Extra Moment *M_E_*

As plotted in Figure 2c, all the beams bend in the *XOY* plane under *M_XY_* and *M_YX_*. With inputs of *M_XY_* and *M_YX_*, the stresses of Areas 1–8 on each beam have the same magnitude but are opposite in directions. The output stresses of Areas 9–16 on the bending neutral layers are almost not influenced by *M_XY_* and *M_YX_*. Thus, with the series connection of strain gauges bounded on the lateral surfaces of each beam, the output variations on Areas 1–8 are counteracted by Circuit X and Y. The effect of *M_XY_* and *M_YX_* on circuit outputs is negligible in theory.

When *M_YZ_* and *M_ZY_* are imposed to the workpiece, the deformation is depicted in Figure 2d. The stresses between Area 10 and 12 or Area 14 and 16 have the same magnitude but are in contrary directions. By ignoring the effect of constraint torsion, the stresses on Areas 9, 11, 13, and 15 are regarded as not affected. On account of the sensor symmetry, when loaded with *M_XZ_* and *M_ZX_*, the stresses on Areas 9–16 are similar to the values produced by *M_YZ_* and *M_ZY_*. Areas 1–8 on the neutral layer of the beams with constraint torsion ignored are not influenced by either of the extra moments in the *X* or *Y* directions. Since all the strain gauges on the top or bottom beam surfaces are serially connected as bridge arms in Circuit Z, the resistance variations on Areas 9–16 are canceled out. There is almost no extra output in each measuring circuit under *M_YZ_*, *M_ZY_*, *M_XZ_*, and *M_ZX_*.

In summary, the output voltages in three measuring circuits are nearly not influenced by *M_E_* in theory.

- Outputs under Eccentric Force *F_A_*

According to the above analysis, and referring to Equations (1)–(4), when an eccentric force *F_A_* is applied to the dynamometer, the output voltages of measuring circuits are expressed below.

(5) $$\{\begin{array}{c} U_{X}(F_{A})=U_{X}(F_{O})+U_{X}(M_{E})=U_{X}(F_{O})=\frac{V_{CC}Kl^{2}}{4REbh(b^{2}+l^{2})}F_{OX}\\ U_{Y}(F_{A})=U_{Y}(F_{O})+U_{Y}(M_{E})=U_{Y}(F_{O})=\frac{V_{CC}Kl^{2}}{4REbh(b^{2}+l^{2})}F_{OY}\\ U_{Z}(F_{A})=U_{Z}(F_{O})+U_{Z}(M_{E})=U_{Z}(F_{O})=-\frac{3V_{CC}Kl}{8REbh^{2}}F_{OZ} \end{array}$$

In view of Equation (5), the three output voltages from Wheatstone bridge circuits caused by eccentric force *F_A_* are converted into the output signals obtained under central force components (*F_OX_*, *F_OY_*, and *F_OZ_*). The extra moments are theoretically inhibited by the combined action of the sensitive area selection in a cross beam and series compensation in measuring circuits.

##### Reduction Method Applied on Developed Sensor

**Figure 4:**
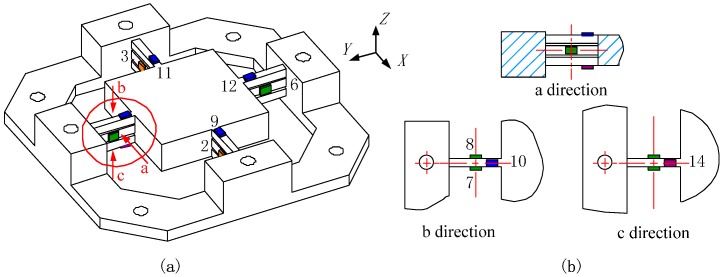
(**a**) Diagram of three-layer cross-beam force sensor; (**b**) partial view of sensitive area adjustment on a three-layer parallel spring element.

To keep coherence with the analysis model of the cross beam described above, and adopt the corresponding *M_E_* reducing method, as well as raise the stiffness of the spring element for milling operation, a dynamometer with three layers of cross beams is proposed, as shown in Figure 4a.

Though the measuring circuits plotted in Figure 3 remain compatible with the developed sensor, the positions of the sensitive regions to bound strain gauges need to be adjusted. Finally, locations of strain gauges on three-layer elastic beams are illustrated in Figure 4. Due to the impact of the boundary constraint, the increased height of the beam in vertical orientation gives rise to a shifting of the neutral layer. The stresses of the sensitive areas assigned to zero are no longer idealized as was mentioned above. The errors of output stresses between the different loading positions are actually cumulative with the growth of the offset between the acting point (Point A) and Point O. Although, compared with the elastic plate as a spring element, the gaps between beam layers are retained to ensure that the boundary constraint is minimal, the output signals from measuring circuits still vary according to the force applied points. Therefore, to evaluate the output errors, the signals must be gauged based on different loading positions.

##### Comparison with Other Fixed Force Sensors

Though there are not enough studies concentrating on the output reduction of extra moment (*M_E_*), according to the simplification of linear relationship between output stress and force or moment applied on an elastic beam, the capacity to prevent the output variation is qualitatively analyzed in Table 1. The case is marked as “Y,” when the extra moment is cancelled by compensation of the measuring circuit or minimized via bonding strain gauge on the neutral layer. If the sensor has no contribution to reducing the moment component, it is symbolized with “N.” A detailed explanation of situations labeled by “N” is attached in Appendix A.

From Table 1, compared with the results in the first two rows, since there is no “N” in this work, the outputs of the additional moments are entirely lessened by the dynamometer proposed in this paper. If one of the circuit outputs is affected, the decoupling result of each force component involves inaccuracy. Therefore, the variation of the sensor output caused by the point where the force is applied changing in this work is smaller than that in the abovementioned fixed-force dynamometers.

### 2.2. Sensor Fabrication

**Figure 5:**
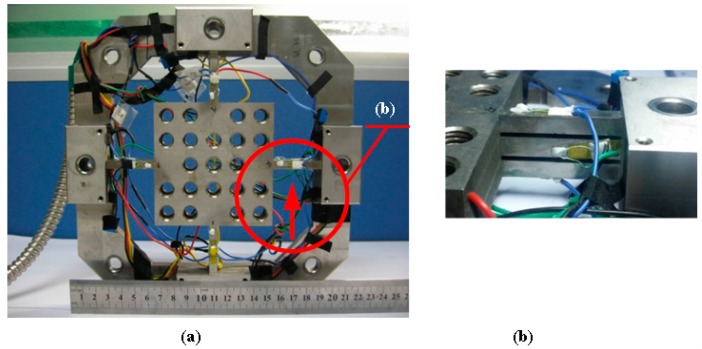
(**a**) Photograph of fabricated sensor; (**b**) the magnified front view of an elastic beam.

As depicted in Figure 5, the three-layer cross-beam force sensor is monolithically made of AISI630 (17–4 PH) stainless steel with elastic modulus (*E*) of 197 GPa and Poisson ratio (*ν*) of 0.272. The size of each single beam for one layer is set as *b* = 6 mm, *h* = 6 mm, *l* = 36 mm, and the height of the slits between two layers is 2 mm. With the center platform having dimensions of 88 mm × 88 mm × 20 mm, the first-order natural frequency achieves 980 Hz via the simulation of Finite Element Method (FEM). TP-3.8-1000 semiconductor strain gauges (produced by Tianguang Sensor, Bengbu, China) are selected to convert stresses into voltage signals, whose resistance values (*R*) are around 1 kΩ. The fixed resistance value (*R_0_*) in the arms of Wheatstone half bridge circuits is 51 kΩ.

## 3. Experimental Results and Discussion

### 3.1. Static Calibration Test

Static calibration test is performed to determine the static relationship between the tri-axial force and the sensor output. To evaluate the positional influence of the loading point on the sensor decoupling performance, the output errors both from desired signals and cross-couplings under eccentric force have to be orderly tested based on the selected points.

**Figure 6:**
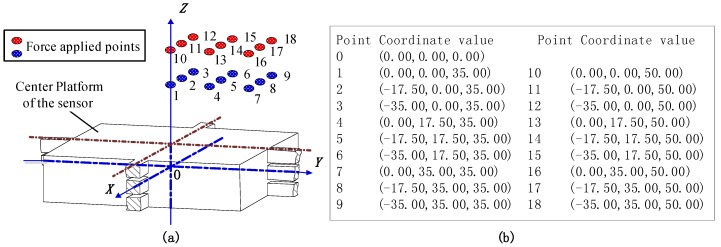
(**a**) The 18 points where force was applied for calibration testing; (**b**) the detailed coordinate values.

**Figure 7:**
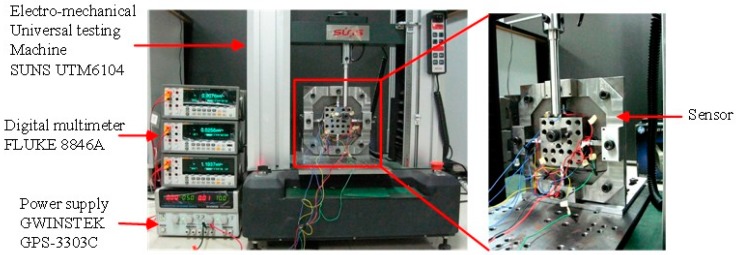
Experimental setup for static calibration.

In view of the symmetrical structure of the sensor, 18 positions from Point 1 to 18 covering 1/4 of the top surface of the center platform were selected to bear unidirectional forces imposed by an electro-mechanical universal testing machine (type UTM6104, SUNS Technology, Shenzhen, China). The geometric center of the central platform is taken as the coordinate origin (Point O). The force acting points are illustrated in Figure 6. The standard forces linearly varied from 0 to 800 N with a step length of 100 N and were separately imposed on the 18 selected positions. All the output voltages in following context are the average values of the signals obtained in loading and unloading procedures under the same load. The static test was performed under a 10 VDC power supply provided by GWINSTEK GPS-3303C, and the output voltages were checked via a digital multimeter, FLUKE 8846A. The schematic view of the experimental setup is shown in Figure 7.

#### 3.1.1. Errors of Desired Signals in Different Positions

**Figure 8:**
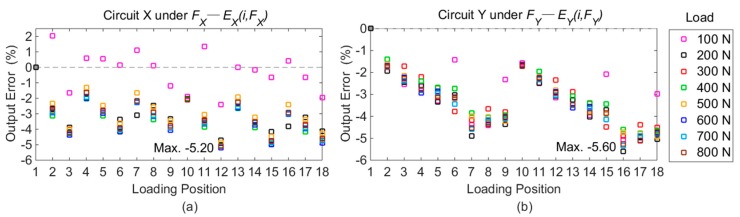

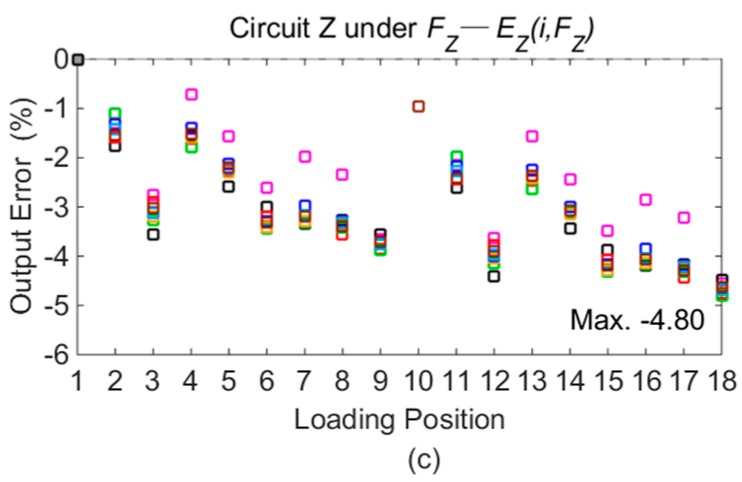
Output errors on different loading points compared with Point 1: (**a**) errors in Circuit X under *F_X_*; (**b**) errors in Circuit Y under *F_Y_*; (**c**) errors in Circuit Z under *F_Z_*.

The output voltages of Point 1 under standard loads are taken as the benchmark. The differences of output voltages between Point 1 and other positions are calculated based on the following equation, and all the fractional errors under each force component are plotted in Figure 8.

(6) $$\begin{array}{ccc} E_{j}(i,F_{j})=\frac{U_{j}(i,F_{j})-U_{j}(1,F_{j})}{U_{j}(1,F_{j})} & i=2,3,...,18; & j=X,Y,Z \end{array}$$

where *E_j_*(*i, F_j_*) is short for the relative error of the desired signal derived on Point *i* in Circuit *j* under unidirectional force *F_j_*; *U_j_*(*) denotes the voltage value measured from Circuit *j* obtained in the calibration test.

As shown in Figure 8, it is apparent that the maximal output deviations in Circuit X, Y, and Z are obtained on Points 12, 16, and 18, respectively, under loads of 600 N, 200 N, and 400 N. The detailed voltage values with maximal differences are listed in Table 2.

As illustrated in Figure 8, the differences of desired signals produced by measured forces (*E_j_(i, F_j_)*) are not more than 5.60%, and the maximum values are caused by positional variations in both the *Y* and *Z* orientations. The results illustrate that the positional influence on the output signals from measured forces is reduced by the developed force sensor.

#### 3.1.2. Variations of Cross-Couplings among Different Positions

**Figure 9:**
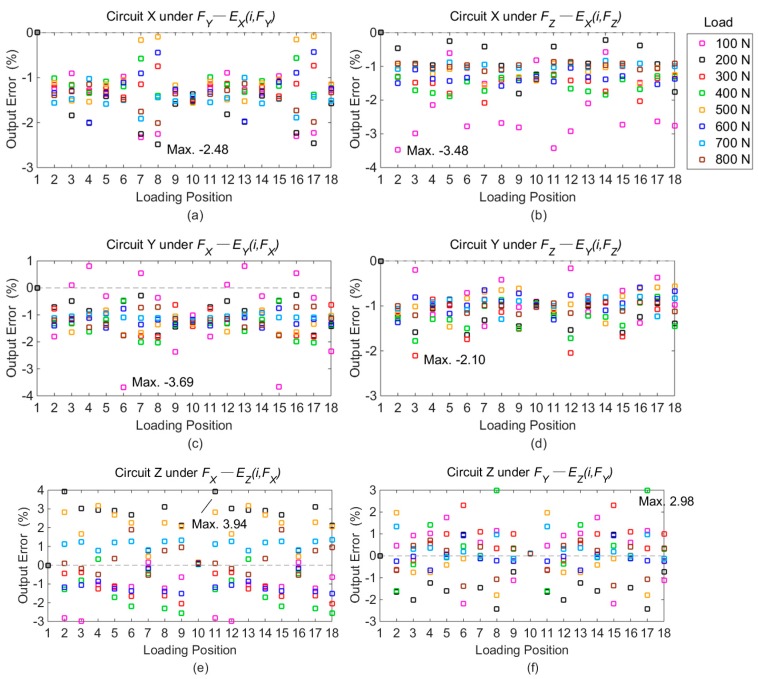
Differences of cross-couplings with output of Point 1 as reference: (**a**) errors in Circuit X under *F_Y_*; (**b**) errors in Circuit X under *F_Z_*; (**c**) errors in Circuit Y under *F_X_*; (**d**) errors in Circuit Y under *F_Z_*; (**e**) errors in Circuit Z under *F_X_*; (**f**) errors in Circuit Z under *F_Y_*.

The cross-coupling could be assessed by the ratio of a signal produced by the interferential force component to the primary output of the same measuring circuit. To facilitate comparison, the differences of the cross interferences are converted using a special ratio referring to the primary output voltages obtained on Point 1, which is expressed in Equation (7). The calculated errors of cross couplings are all depicted in Figure 9.

(7) $$\begin{array}{ll} E_{j}(i,F_{k})=\frac{U_{j}(i,F_{k})-U_{j}(1,F_{k})}{U_{j}(1,F_{j})} & i=1,2,3,....,18\\ j=X,Y,Z; & k=X,Y,Z;\;j\neq k \end{array}$$

where *E_j_*(*i, F_k_*) stands for the error of cross coupling obtained in Circuit *j*, when *F_k_* is applied on Point *i*, and it takes the voltage value gauged in Circuit *j* and generated by *F_j_* on Point 1 (*U_j_*(*1, F_j_*)) as reference.

As shown in Figure 9, the maximal deviations of six cross interferences separately appear with different loads and positions. The concrete conditions are given in Table 3.

As plotted in Figure 9, the deviations of cross couplings among different loading points (*E_j_(i, F_k_)*) are within 3.94%, and the maximal difference is brought by the resultant displacement in *X* and *Z* directions. The results indicate that the positional effect on the output voltages of cross interferences is also decreased by the developed dynamometer.

#### 3.1.3. Static Decoupling Matrix

**Figure 10:**
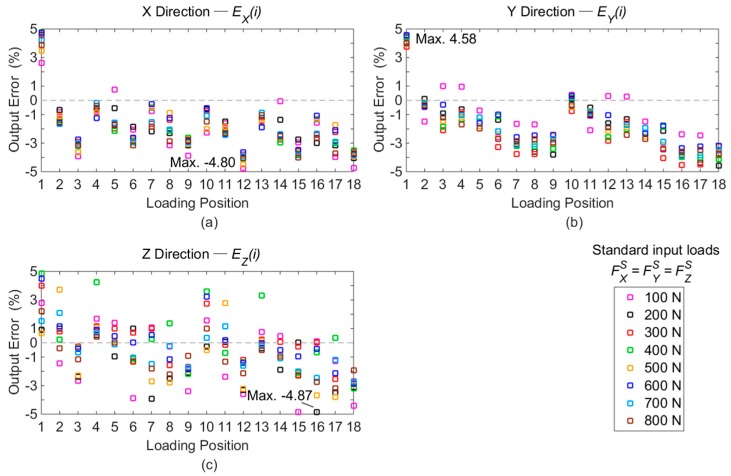
Errors of force solutions compared with standard force inputs: (**a**) errors of *F_X_*; (**b**) errors of *F_Y_*; (**c**) errors of *F_Z_*.

Finally, a decoupling matrix (*D*) is derived from the static calibration and given in Equation (8). In order to evaluate the decoupling performance, input loads with three equal force components are taken as examples. The measured data gauged from 100 to 800 N in calibration test are all involved to simplify the experimental procedure. On each loading point, three orthogonal forces ($F_{X}^{S},F_{Y}^{S}$, and $F_{Z}^{S}$) with the same magnitude imposed by universal testing machine are combined together as one standard load, and the superposition of the corresponding output signals based on measuring circuits (Circuit X, Y, and Z) are considered as the output voltages (*U_X_*, *U_Y_* and *U_Z_*) obtained under the standard input. According to Equation (8), 18 × 8 groups of force solutions containing *F_X_*, *F_Y_*, and *F_Z_* are obtained based on the decoupling matrix (*D*) and output values (*i.e.*, *U_X_*, *U_Y_* and *U_Z_*). Compared with the standard loads, the errors of the solutions are calculated based on Equation (9) and plotted in Figure 10.

(8) $$\begin{array}{l} U_{OUT}=DF+U^{Z}\\ \left[\begin{array}{c} U_{X}\\ U_{Y}\\ U_{Z} \end{array}\right]=\left[\begin{array}{ccc} 1.72\times 10^{-2} & -6.94\times 10^{-4} & -1.03\times 10^{-3}\\ -6.00\times 10^{-4} & 1.71\times 10^{-2} & -1.05\times 10^{-3}\\ -6.26\times 10^{-3} & -5.83\times 10^{-3} & 1.25\times 10^{-1} \end{array}\right]\left[\begin{array}{c} F_{X}\\ F_{Y}\\ F_{Z} \end{array}\right]+\left[\begin{array}{c} 6\text{.25}\times 10^{-1}\\ 5.38\times 10^{-1}\\ 3.23\times 10^{-1} \end{array}\right] \end{array}$$

where *U_OUT_* is the matrix of output voltages involving three-circuit outputs (*U_X_*, *U_Y_* and *U_Z_*), and the unit is mV; *U^Z^* includes the zero offsets of the three measuring circuits, and its unit is also mV.

(9) $$\begin{array}{c} E_{j}(i)=\frac{F_{j}(i)-F_{j}^{S}}{F_{j}^{S}}\;i=1,2,3,....,18;\;j=X,Y,Z\\ F_{X}^{S}=F_{Y}^{S}=F_{Z}^{S}=100\;N,200\;N,300\;N,....,800\;N \end{array}$$

where *E_j_(i)* is defined as the error between the standard input ($F_{j}^{S}$) and each solved force component (*F_j_*(*i*)).

The maximal differences of each force component are clearly gained as depicted in Figure 10. The entire force solutions with the maximal deviations marked in Figure 10 are tabulated in Table 4.

As presented in Figure 10, the deviations of the measured force components among the calibration positions are not larger than 4.87%. On account of the maximal differences between the standard forces and the measured values, the static deviations are acceptable when the force applied point is moving within calibration range.

### 3.2. Milling Experiment

An application of the sensor in the milling process was carried out to verify its dynamic performance in the actual machining operation.

#### 3.2.1. Measuring Range Estimation

To ensure the milling accuracy, the deflection of the bearing table under the workpiece has to be addressed [17]. In order to keep the sensor center platform within modest displacement, the fixed dynamometer requires a limit in its measuring range. When the deformation of the center platform is considered, its own shape change must be taken into account. When an AISI1045 steel workpiece of 88 mm × 88 mm × 15 mm is placed on the sensor, with the force applied point chosen at the vertex of the corner of the workpiece, the maximal deformation of the sensor center table is obtained. In static FEM (Finite Element Method) simulation, when 800 N of *F_X_* is imposed, the *X*, *Y*, and *Z* directional displacements are not more than 1.09 × 10^−2^ mm, 8.29 × 10^−3^ mm, and 6.98 × 10^−3^ mm; loaded with 400 N of *F_Z_*, the maximal deformations in the *Z*, *X*, and *Y* directions are 2.00 × 10^−2^ mm, 1.36 × 10^−3^ mm, and 1.36 × 10^−3^ mm. Therefore, according to sensor symmetry, the measuring range of the developed dynamometer is set as from −800 N to 800 N for *F_X_*, from −800 N to 800 N for *F_Y_*, and from −400 N to 400 N for *F_Z_*. Based on the measuring range, vertical milling was selected in the following milling test.

#### 3.2.2. Natural Frequency Identification

**Figure 11:**
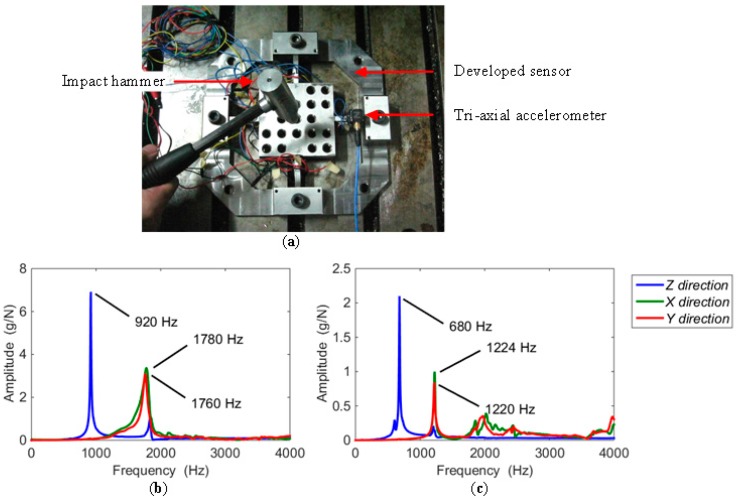
(**a**) Impact testing for the developed sensor; (**b**) frequency and amplitude results of sensor impact testing; (**c**) impact test results with a workpiece.

In a milling experiment, the testing frequency is caused by the edge of the tool cutting into the workpiece. The identification of natural frequency must be achieved before the milling experiment. With the dynamometer mounted on the milling table, the measurement of natural frequencies was performed via hammer impact testing. As shown in Figure 11a, with a tri-axial accelerometer (type 95663) attached on the beam, the sensor was tapped by the impact hammer (type 086D05) at a chosen exciting point, and in one direction at a time. The signals of force and acceleration separately derived from the impact hammer and accelerometer were both collected in a SIEMENS mobile LMS SCADAS305 data acquisition cabinet, and processed by the software of LMS Test Lab. The natural frequencies of the sensor in the *X*, *Y*, and *Z* directions are 1780 Hz, 1760 Hz, and 920 Hz, as depicted in Figure 11b. To complete the following milling test, when a workpiece of 88 mm × 88 mm × 15 mm made by AISI1045 steel was fastened by four M10 hexagonal screws onto the center platform, the natural frequencies were also measured. The results are plotted in Figure 11c, in which the abscissa values corresponding to the curve peaks in the *X*, *Y*, and *Z* directions are 1224 Hz, 1220 Hz, and 680 Hz, respectively. To avoid exciting vibration affecting the precision of the sensor, it is suggested that the lowest natural frequency of the sensor should be at least four times larger than the exciting frequency [18]. Thus, in a milling test, supposing a cutter with one flute is used, the highest spindle speed has to be lower than 680 Hz/4 = 170 Hz = 10,200 rpm. When a milling tool with three cutting edges is selected in an experiment, the spindle speed does not exceed 680/4/3 ≈ 56.67 Hz = 3400 rpm. Due to the gaps retained on the sensor beams, though the natural frequency of the dynamometer is enough for milling experiment, it is still inadequate for actual application. The stiffness enhancement still requires further research.

#### 3.2.3. Dynamic Milling Test

**Figure 12:**
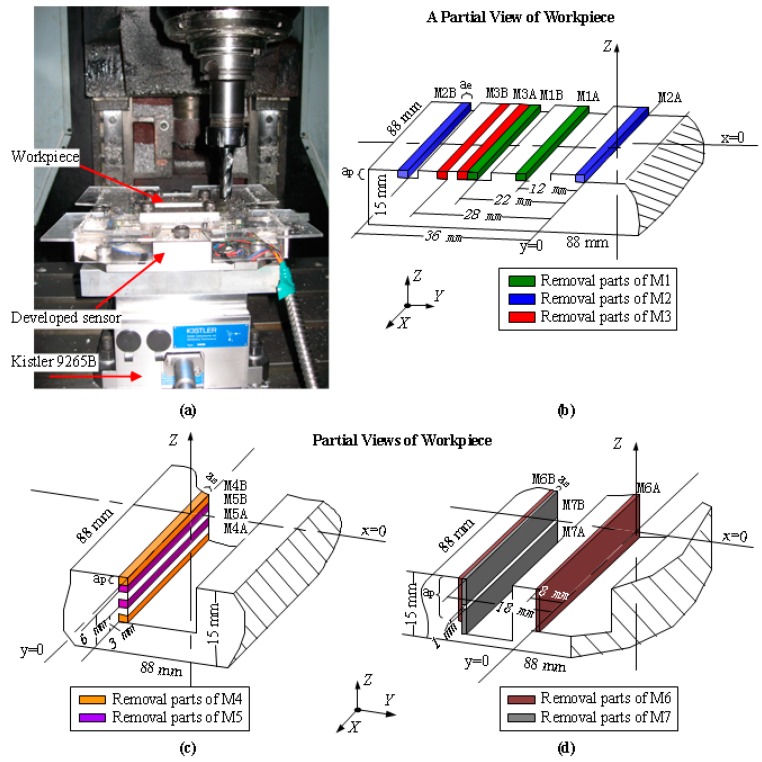
(**a**) A photograph of the milling experiment; (**b**) a partial view of the workpiece for M1–M3; (**c**) a partial view of the workpiece for M4 and M5; (**d**) a partial view of the workpiece for M6 and M7.

Dynamic milling testing was carried out by peripheral milling on AISI1045 steel workpieces, with three-edge solid carbide end milling cutters (manufactured by AHNO Tool, Suzhou, China), whose diameters are 16 mm. The experiments were operated in a BCH850 three-axes CNC milling machine under dry cutting conditions. Cooling time was necessary during the experiment to minimize the temperature variation and restrain the corresponding error caused to the sensor output. As plotted in Figure 12b–d, seven pairs of milling positions (M1–M7) were chosen to machine step shapes via up-milling with the feeding direction along the negative *X* axis. In each group, two different places were involved to assess the influence of the cutting positions, are distinguished by suffixes A and B designated in their names (e.g., M1A and M1B in group M1). In testing groups M1–M5, a milling cutter with helix angle of 15° was used, and machining parameters were set as follows: radial cutting depth (*a_e_*) of 2 mm, axial depth (*a_p_*) of 1.5 mm, and feed rate (*f_z_*) of 1/12 mm per tooth. Spindle speeds (n) of 600 rpm, 2000 rpm, 3000 rpm, 1000 rpm, and 2500 rpm, respectively, were tested at positions M1–M5. To validate the positional influence under larger values of *F_Z_*, a milling tool with helix angle of 45° was employed in experimental groups M6 and M7. Machining parameters were configured as cutting depth (*a_e_*) of 1 mm and feed rate (*f_z_*) of 0.15 mm per tooth. In M6 and M7, axial depths (*a_p_*) were separately selected as 10 mm and 6 mm, and spindle speeds (*n*) were chosen as 500 rpm and 1500 rpm, respectively. TI PGA308 chips were used to amplify the sensor output signals within 0–5 V, and the waveforms were detected by an oscilloscope, Tektronix MSO 4104, configured with a sampling rate of 10 KHz. As shown in Figure 12a, the sensor was placed on a Kistler 9265B dynamometer, which was employed as a reference of standard force value. Since the length of the sensor pedestal is longer than the width of the standard dynamometer, both of their natural frequencies are reduced, and additional vibration was brought into the output signals. Hence, a program of 600 Hz low-pass pre-filter was introduced before data processing.

**Figure 13:**
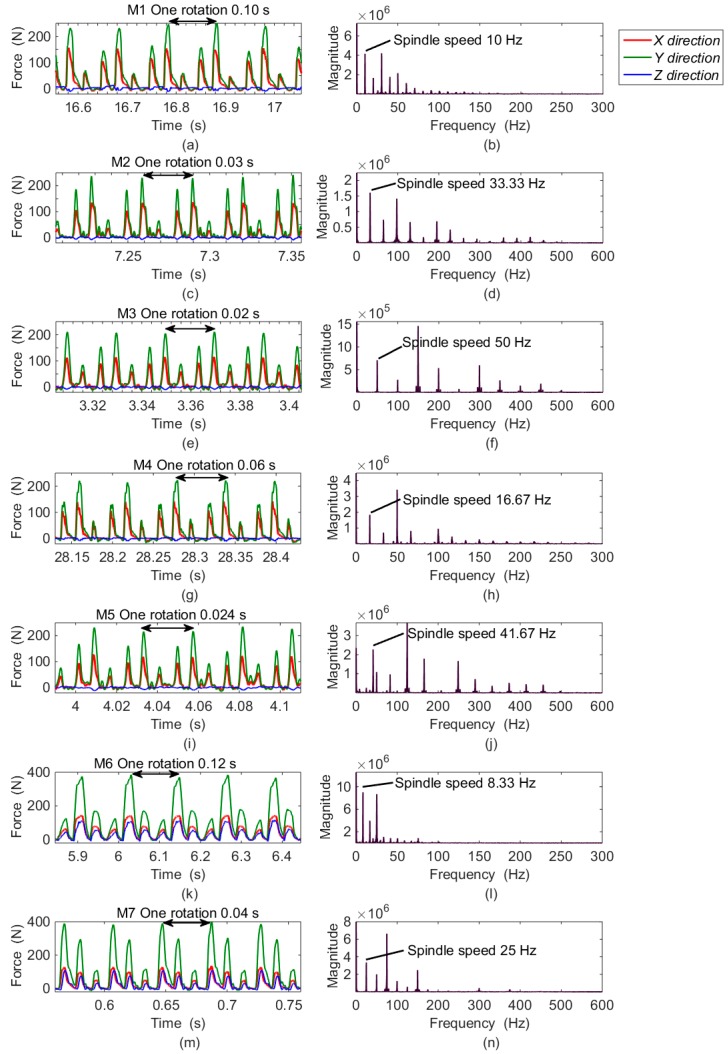
The partial enlarged view of milling forces in different positions: (**a**) forces of M1B in the time domain; (**b**) *F_X_* of M1B in the frequency domain; (**c**) forces of M2B in the time domain; (**d**) *F_X_* of M2B in the frequency domain; (**e**) forces of M3B in the time domain; (**f**) *F_X_* of M3B in the frequency domain; (**g**) forces of M4B in the time domain; (**h**) *F_X_* of M4B in the frequency domain; (**i**) forces of M5B in the time domain; (**j**) *F_X_* of M5B in the frequency domain; (**k**) forces of M6B in the time domain; (**l**) *F_X_* of M6B in the frequency domain; (**m**) forces of M7B in the time domain; (**n**) *F_X_* of M7B in the frequency domain.

The recorded waveforms in the time domain obtained at M1B–M7B are plotted in Figure 13, and the corresponding Fourier transformations of *F_X_* are depicted on the right side. Because of the similarity, the data for subgroup A and Fourier transformations of *F_Y_* and *F_Z_* are all skipped in the graph. The milling periods are clearly demonstrated both from the peak repetition in the time domain and the component distributions in the frequency domain.

**Figure 14:**
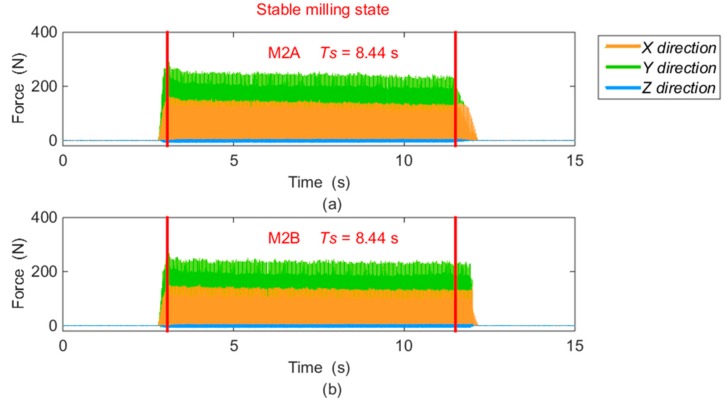
Milling forces from M2 in stable state: (**a**) data from M2A; (**b**) data from M2B.

In order to compare the measured results between different milling positions, the data in the same pair with an equal amount of time (*T_S_*) are extracted from the measured force values in the stable milling state, as the example of shrinking views for M2 shown in Figure 14. Based on the selected data, the peak to peak amplitudes of each force component (*F_X_*, *F_Y_*, and *F_Z_*) gained from every spindle cycle are averaged. The computational method is expressed in Equation (10), and the solved results are filled in Table 5.

(10) $$\begin{array}{ccc} F_{j}{\left(i\right)}_{P-P}=\frac{1}{N}\sum_{c=1}^{N}F_{j}{\left(i,c\right)}_{P-P}=\frac{1}{T_{S}n}\sum_{c=1}^{T_{S}n}F_{j}{\left(i,c\right)}_{P-P} & i=M1,M2,M3,...,M7; & j=X,Y,Z \end{array}$$

where *N* represents the number of the spindle cycles involved in the period of *T_S_*; *F_j_(i, c)_P-P_* denotes the peak to peak amplitude derived in the *c-*th spindle cycle from the position of M1–M7; and *F_j_*(*i*)*_P-P_* is the average value of peak to peak force amplitude separately obtained in M1–M7.

In Table 5, the measured forces derived from the static decoupling matrix (Equation (8)) are compared with the benchmarks obtained from the standard dynamometer. The coefficient is the quotient obtained by measured value dividing its reference. The ratios are 1.36% ± 4.51%, 1.33% ± 2.34%, and 1.51% ± 5.99%, respectively, in the *X*, *Y*, and *Z* directions, which proves the validity of the measured data compared with the output signals derived from the standard dynamometer.

Based on Table 5, the output variations in different milling groups are expressed via fractional errors, and subgroup A is taken as the reference. The calculation formula is given in Equation (11), and the results are tabulated in Table 6.

(11) $$\begin{array}{c} E_{j}{\left(i\right)}_{P-P}=\frac{F_{j}{\left(i\right)}_{P-P}-F_{j}{\left(k\right)}_{P-P}}{F_{j}{\left(k\right)}_{P-P}}\;j=X,Y,Z\\ \;when\;i=dB,k=dA,d=M1,M2,M3,...,M7 \end{array}$$

where *E_j_*(*i*)*_P-P_* represents the output deviation between the two average peak to peak force values derived in each pair.

As illustrated in Table 6, roughly compared by subtraction, the maximal deviation between tested and standard force variation is 6.61%, which appears in the *Z* direction. Due to the lowest stiffness being along the *Z* axis, it is most easily affected by the additional vibration caused by the sensor installation, as mentioned above. Although the pre-filter has been used, the force amplitude is still impacted, which also makes the *F_Z_* coefficient deviation listed in Table 5 larger than the other two ratios derived from *F_X_* and *F_Y_*. Moreover, though a cooling period has been taken, due to the different distances between strain gauges and milling positions, the influence of temperature changing was not completely eliminated by the measuring circuit. Considering these issues, the deviations of the measured forces are acceptable in the range of the center platform for a monitoring system applied in the milling process.

## 4. Conclusions

In order to decrease sensor output errors brought by different loading positions in dynamic milling process, a tri-axial milling dynamometer based on three-layer cross beams is proposed in this paper. With the special consideration of sensor structure selection, sensitive area designation, and measuring circuit establishing, the influence on sensor outputs caused by eccentric milling force is reduced.

A comparison of test results under the same load input and operated in different parts of the workpiece was taken. With standard forces imposed in a static calibration test, the maximal deviation between the measured forces and the standard inputs is 4.87%. During a dynamic milling test, measured forces derived with identical machining parameters in different positions are compared. By taking a commercial sensor as reference, the differences of milling force variations are not more than 6.61%. Experimental results demonstrate that the developed dynamometer is appropriate for dynamic force measurement when a milling tool cuts in different places on the workpiece, which has potential application value for milling force measurement in a monitoring system.

In this work, the measuring range and milling testing result are both affected by the stiffness of the sensor, which requires further improvement. The structure optimization and combination based on cross beam have to be done in the next step. Future work will also focus on an optimal solution for sensor decoupling, for example, reducing the error in a certain direction to extract the main force more accurately in different milling modes during the machining process.

## Figures and Tables

**Table 1:** The comparison of extra moment interference between different sensor structures.

Sensors		*F_AX_*						*F_AY_*						*F_AZ_*					
	Inputs	*M_XY_*			*M_XZ_*			*M_YX_*			*M_YZ_*			*M_ZX_*			*M_ZY_*		
	Output Circuits	X	Y	Z	X	Y	Z	X	Y	Z	X	Y	Z	X	Y	Z	X	Y	Z
[11,12,13,14]		Y	Y	N	N	Y	N	Y	Y	N	Y	N	N	N	Y	N	Y	N	N
[15]		Y	N	Y	N	Y	N	Y	N	Y	N	Y	Y	N	Y	N	N	Y	Y
This work		Y	Y	Y	Y	Y	Y	Y	Y	Y	Y	Y	Y	Y	Y	Y	Y	Y	Y

**Table 2:** Maximal deviations of desired signals in different positions.

Max. Deviation Conditions	*F_X_* in Circuit X		*F_Y_* in Circuit Y		*F_Z_* in Circuit Z	
Force inputs (N)	*F_X_* = 600		*F_Y_* = 200		*F_Z_* = 400	
Point numbers *i*	1	12	1	16	1	18
Outputs (mV)	10.72	10.17	3.66	3.46	25.70	24.44
Max. deviations (%)	*E_X_*(12, 600) = −5.20		*E_Y_*(16, 200) = −5.60		*E_Z_*(18, 400) = −4.80	

**Table 3:** Maximal deviations of cross couplings in different positions.

Max. Deviation Conditions	*F_X_* in Circuit X	*F_Y_* in Circuit X		*F_X_* in Circuit X	*F_Z_* in Circuit X		*F_Y_* in Circuit Y	*F_X_* in Circuit Y	
Force inputs (N)	*F_X_* = 200	*F_Y_* = 200		*F_X_* = 100	*F_Z_* = 100		*F_Y_*= 100	*F_X_* = 100	
Point numbers *i*	1	1	8	1	1	2	1	1	1
Outputs (mV)	3.70	0.12	0.03	1.90	0.16	0.09	1.92	0.14	0.07
Max. deviations (%)	*E_X_*(8, 200) = −2.48			*E_X_*(2, 100) = −3.48			*E_Y_*(6, 100) = −3.69		
**Max. Deviation Conditions**	***F_Y_* in Circuit Y**	***F_Z_* in Circuit Y**		***F_Z_* in Circuit Z**	***F_X_* in Circuit Z**		***F_Z_* in Circuit Z**	***F_Y_* in Circuit Z**	
Force inputs (N)	*F_Y_* = 300	*F_Z_* = 300		*F_Z_* = 200	*F_X_* = 200		*F_Z_* = 400	*F_Y_* = 400	
Point numbers *i*	1	1	3	1	1	11	1	1	17
Outputs (mV)	5.39	−0.08	−0.19	25.70	−1.71	−0.70	51.31	−2.56	−1.03
Max. deviations (%)	*E_Y_*(3, 300) = −2.10			*E_Z_*(11, 200) = 3.94			*E_Z_*(17, 400) = 2.98		

**Table 4:** The maximum deviations of solved forces in different positions.

Max. Deviation Conditions		*X* Direction	*Y* Direction	*Z* Direction
Standard inputs (N)		$F_{X}^{S}=F_{Y}^{S}=F_{Z}^{S}$ = 100	$F_{X}^{S}=F_{Y}^{S}=F_{Z}^{S}$ = 600	$F_{X}^{S}=F_{Y}^{S}=F_{Z}^{S}$ = 200
Point numbers *i*		12	1	16
Force solutions (N)	*F_X_*	95.20	628.46	194.05
	*F_Y_*	100.29	627.50	192.33
	*F_Z_*	96.39	626.86	190.26
Deviations (%)	*F_X_*	*E*(12, 100) = −4.80 *	*E*(1, 600) = 4.74	*E*(16, 200) = −2.98
	*F_Y_*	*E*(12, 100) = 0.29	*E*(1, 600) = 4.58 *	*E*(16, 200) = −3.83
	*F_Z_*	*E*(12, 100) = −3.61	*E*(1, 600) = 4.48	*E*(16, 200) = −4.87 *

* The items are the maximum differences in each direction.

**Table 5:** The averaged peak to peak values of milling force in each group.

Pairs		Time *T_S_* (s)	Average Peak to Peak Values of *F_X_* (N)		Average Peak to Peak Values of *F_Y_* (N)		Average Peak to Peak Values of *F_Z_* (N)	
			Developed Sensor	Standard Sensor	Developed Sensor	Standard Sensor	Developed Sensor	Standard Sensor
M1	A	33.90	157.51	208.16	248.15	315.81	17.48	25.52
	B		153.84	206.73	246.10	318.15	18.43	26.23
	A		Coefficient	1.32	Coefficient	1.28	Coefficient	1.46
	B			1.34		1.28		1.42
M2	A	8.44	143.14	195.14	247.01	313.01	14.13	22.68
	B		137.90	192.31	238.57	319.95	14.02	21.46
	A		Coefficient	1.36	Coefficient	1.27	Coefficient	1.61
	B			1.39		1.34		1.53
M3	A	6.86	126.73	174.26	229.01	307.08	12.45	19.81
	B		121.99	173.44	223.46	304.19	13.15	20.01
	A		Coefficient	1.38	Coefficient	1.34	Coefficient	1.59
	B			1.42		1.36		1.52
M4	A	20.12	149.90	199.16	251.60	322.18	15.31	23.40
	B		156.78	204.83	242.71	318.81	14.39	22.52
	A		Coefficient	1.33	Coefficient	1.28	Coefficient	1.53
	B			1.31		1.31		1.57
M5	A	7.66	130.07	176.90	232.76	308.70	12.79	21.60
	B		124.05	172.41	227.22	306.07	12.41	21.10
	A		Coefficient	1.36	Coefficient	1.33	Coefficient	1.56
	B			1.39		1.35		1.61
M6	A	22.28	172.31	223.86	384.22	492.40	116.92	179.45
	B		162.84	223.23	389.77	517.00	124.74	179.95
	A		Coefficient	1.30	Coefficient	1.28	Coefficient	1.53
	B			1.37		1.33		1.44
M7	A	6.80	129.01	169.01	368.46	488.07	114.39	181.30
	B		127.66	174.35	381.61	483.22	117.56	182.01
	A		Coefficient	1.31	Coefficient	1.32	Coefficient	1.59
	B			1.37		1.27		1.55

**Table 6:** The differences of the averaged peak to peak values of milling force in each group.

Positions	Differences *E_X_(i)_P-P_* (%)		Differences *E_Y_(i)_P-P_* (%)		Differences *E_Z_(i)_P-P_* (%)	
	Developed Sensor	Standard Sensor	Developed Sensor	Standard Sensor	Developed Sensor	Standard Sensor
M1	−2.33	−0.69	−0.82	0.74	5.44	2.79
	Error (%)	−1.64	Error (%)	−1.56	Error (%)	2.65
M2	−3.66	−1.45	−3.42	2.22	−0.79	−5.37
	Error (%)	−2.21	Error (%)	−5.64	Error (%)	4.58
M3	−3.74	−0.47	−2.43	−0.94	5.60	−1.01
	Error (%)	−3.27	Error (%)	−1.49	Error (%)	6.61
M4	4.59	2.85	−3.53	−1.05	−6.01	−3.74
	Error (%)	1.74	Error (%)	−2.48	Error (%)	−2.27
M5	−4.63	−2.54	−2.38	−0.85	−5.32	−2.33
	Error (%)	−2.09	Error (%)	−1.53	Error (%)	−2.99
M6	−5.50	−0.28	1.45	5.00	6.69	0.28
	Error (%)	−5.22	Error (%)	−3.55	Error (%)	6.41
M7	−1.05	3.16	3.57	−0.99	2.77	0.39
	Error (%)	−4.21	Error (%)	4.56	Error (%)	2.38

**Figure A1:**
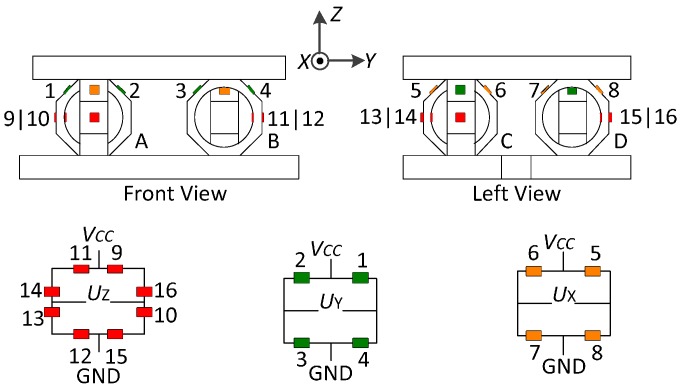
A diagram of the fixed force sensor.

**Figure A2:**
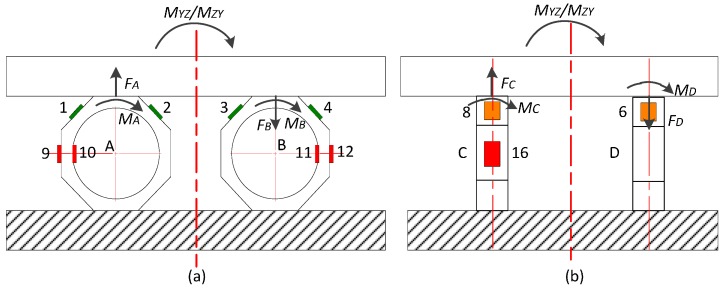
(**a**) Ring A and B under *X* axial moment; (**b**) Ring C and D under *X* axial moment.

**Figure A3:**
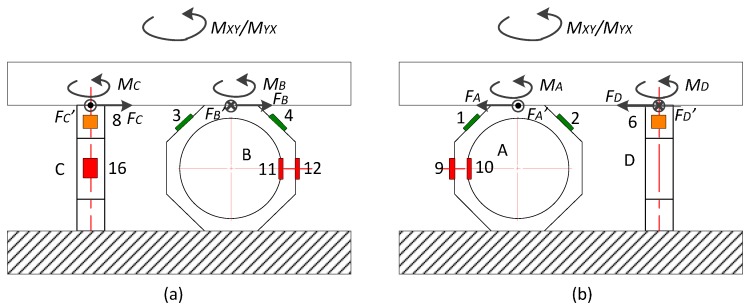
(**a**) Ring B and C under *Z* axial moment; (**b**) Ring A and D under *Z* axial moment.

**Figure A4:**
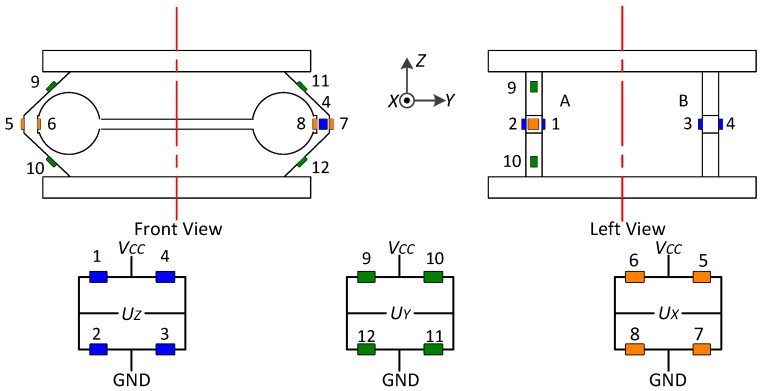
A diagram of the fixed force sensor.

**Figure A5:**
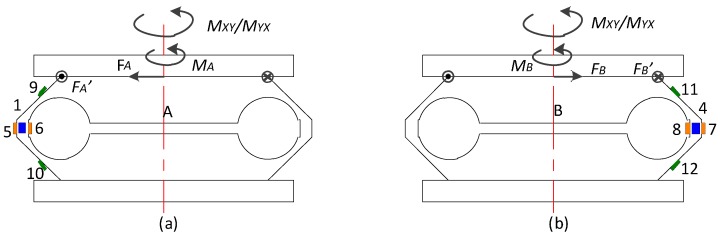
(**a**) Elastic Beam A under *Z* axial moment; (**b**) Elastic Beam B under *Z* axial moment.

**Figure A6:**
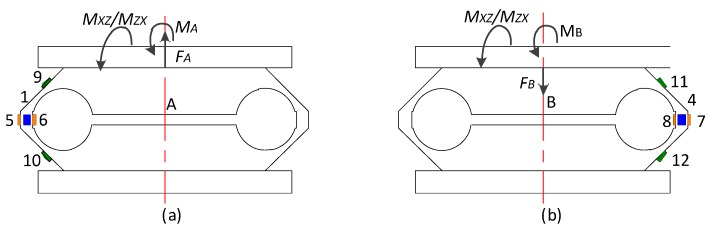
(**a**) Elastic Beam A under *Y* axial moment; (**b**) Elastic Beam B under *Y* axial moment.

**Figure A7:**
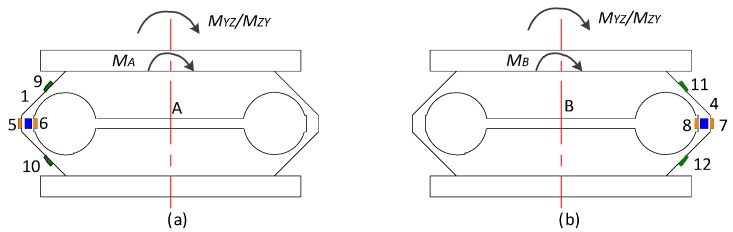
(**a**) Elastic Beam A under *X* axial moment; (**b**) Elastic Beam B under *X* axial moment.

## Appendix A

The explanation only focuses on the situations with “N” in Table 1. If the output of the measuring circuit is independent of the extra moment component, its output voltage must be zero, otherwise the sensor is sensitive to the movement of the point where force is applied. All the axial stress caused by constraint torsion is ignored in the following context.

### A.1. Influence of M_E_ on Sensor in [11,12,13,14]

The force sensor is plotted in Figure A1. In considering the sensor symmetry between the *X* and *Y* directions, only the outputs of measuring circuits under *X* and *Z* axial moments (*M_YZ_*, *M_ZY_*, *M_XY_* and *M_YX_*) need to be discussed.

#### A.1.1. *M_YZ_* and *M_ZY_* Applied on the Sensor

Loaded with *M_YZ_* and *M_ZY_*, the force analysis of the sensor is illustrated in Figure A2. In elastic range, by ignoring non-linear deformation, the output stress of an octagonal ring is directly proportional to its input force (*F_A_*–*F_D_*) or moment (*M_A_*–*M_D_*). The tangential stresses (*σ_1_*–*σ_4_* and *σ_9_*–*σ_16_*) at the center point of Areas 1–4 and 9–16 are expressed as follows. According to the sensor symmetry, the elements in coefficient matrices are further simplified.

(A1) $$\{\begin{array}{l} \left[\begin{array}{c} \sigma_{1}\\ \sigma_{2} \end{array}\right]=\left[\begin{array}{cc} -C_{1} & C_{1}’\\ -C_{2} & -C_{2}’ \end{array}\right]\left[\begin{array}{c} F_{A}\\ M_{A} \end{array}\right]=\left[\begin{array}{cc} -C_{1} & C_{1}’\\ -C_{1} & -C_{1}’ \end{array}\right]\left[\begin{array}{c} F_{A}\\ M_{A} \end{array}\right]\\ \left[\begin{array}{c} \sigma_{3}\\ \sigma_{4} \end{array}\right]=\left[\begin{array}{cc} C_{3} & C_{3}’\\ C_{4} & -C_{4}’ \end{array}\right]\left[\begin{array}{c} F_{B}\\ M_{B} \end{array}\right]=\left[\begin{array}{cc} C_{1} & C_{1}’\\ C_{1} & -C_{1}’ \end{array}\right]\left[\begin{array}{c} F_{A}\\ M_{A} \end{array}\right] \end{array}$$

(A2) $$\{\begin{array}{l} \left[\begin{array}{c} \sigma_{9}\\ \sigma_{10} \end{array}\right]=\left[\begin{array}{cc} -C_{9} & -C_{9}’\\ C_{10} & C_{10}’ \end{array}\right]\left[\begin{array}{c} F_{A}\\ M_{A} \end{array}\right]=\left[\begin{array}{cc} -C_{9} & -C_{9}’\\ C_{10} & C_{10}’ \end{array}\right]\left[\begin{array}{c} F_{A}\\ M_{A} \end{array}\right]\\ \left[\begin{array}{c} \sigma_{11}\\ \sigma_{12} \end{array}\right]=\left[\begin{array}{cc} -C_{11} & -C_{11}’\\ C_{12} & C_{12}’ \end{array}\right]\left[\begin{array}{c} F_{B}\\ M_{B} \end{array}\right]=\left[\begin{array}{cc} -C_{10} & -C_{10}’\\ C_{9} & C_{9}’ \end{array}\right]\left[\begin{array}{c} F_{A}\\ M_{A} \end{array}\right]\\ \left[\begin{array}{cc} \sigma_{13} & \sigma_{14} \end{array}\right]=\left[\begin{array}{cc} C_{13} & -C_{14} \end{array}\right]F_{D}=\left[\begin{array}{cc} C_{9} & -C_{10} \end{array}\right]F_{A}\\ \left[\begin{array}{cc} \sigma_{15} & \sigma_{16} \end{array}\right]=\left[\begin{array}{cc} C_{15} & -C_{16} \end{array}\right]F_{C}=\left[\begin{array}{cc} C_{10} & -C_{9} \end{array}\right]F_{A} \end{array}$$

where *C_1_-C_4_* and *C_9_-C_16_* denote to the flexibility coefficient between forces and stresses; *C_1_’-C_4_’* and *C_9_’-C_16_’* are the coefficient elements corresponding to input moments.

According to the measuring circuits illustrated in Figure A1, the output voltages of *U_Y_* and *U_Z_* under *M_YZ_* or *M_ZY_* are calculated below based on Equations (A1) and (A2). *R* is the resistance value of each strain gauge bounded on the sensitive areas, and *ΔR* is the variation of *R*.

(A3) $$\begin{array}{cc} U_{Y} & =\left(\frac{R_{2}}{R_{2}+R_{3}}-\frac{R_{1}}{R_{1}+R_{4}}\right)V_{CC}=\left(\frac{R+\Delta R_{2}}{2R+\Delta R_{2}+\Delta R_{3}}-\frac{R+\Delta R_{1}}{2R+\Delta R_{1}+\Delta R_{4}}\right)V_{CC}\\ & =-\frac{K\times M_{A}\times C_{1}’}{R}V_{CC} \end{array}$$

(A4) $$\begin{array}{cc} U_{Z} & =\left(\frac{R_{11}+R_{14}}{R_{11}+R_{12}+R_{13}+R_{14}}-\frac{R_{9}+R_{16}}{R_{9}+R_{10}+R_{15}+R_{16}}\right)V_{CC}=\left(\frac{2R+\Delta R_{11}+\Delta R_{14}}{4R+\sum_{i=11}^{14}\Delta R_{i}}-\frac{2R+\Delta R_{9}+\Delta R_{16}}{4R+\sum_{i=9}^{10}\Delta R_{i}+\sum_{i=15}^{16}\Delta R_{i}}\right)V_{CC}\\ & =\begin{array}{c} (\frac{2R-K\times \left[M_{A}\times C_{10}’+2F_{A}\times C_{10}\right]}{4R+K\times \left[M_{A}\times \left(C_{9}’-C_{10}’\right)+2F_{A}\times (C_{9}-C_{10})\right]}-\\ \frac{2R-K\times \left[M_{A}\times C_{9}’+2F_{A}\times C_{9}\right]}{4R-K\times \left[M_{A}\times \left(C_{9}’-C_{10}’\right)+2F_{A}\times (C_{9}-C_{10})\right]})V_{CC} \end{array} \end{array}$$

where *K* is the working sensitivity coefficient of strain gauges; *V_CC_* is the supply voltage of measuring circuit.

Due to the different curvature of the inner and outer surfaces in an octagonal ring, *C_10_*, *C_10_’* on the internal race are not equal to *C*_9_, *C*_9_’ on the external plane. From Equations (A3) and (A4), it is obvious that the output signals of *U_Y_* and *U_Z_* are affected by *F_A_* and *M_A_*, which is caused by the tilt and constraint of the upper plate. Thus, a sensor with octagonal rings is not able to cancel out the effect of *M_YZ_* and *M_ZY_*.

Based on the sensor symmetry, *U_Y_* and *U_Z_* are also impacted by *M_XZ_* and *M_ZX_*.

#### A.1.2. *M_XY_* and *M_YX_* Applied on the Sensor

Loaded with *M_XY_* and *M_YX_*, the force analysis of the sensor is illustrated in Figure A3. Affected by the upper plate displacement, input force (*F_A_*–*F_D_* and *F_A_’*–*F_D_’*) and moment (*M_A_*–*M_D_*) are imposed to each top surface of the rings. The relationship between the input values and output tangential stresses (*σ_9_*–*σ_16_*) are listed below:

(A5) $$\{\begin{array}{l} \left[\begin{array}{cc} \sigma_{9} & \sigma_{10} \end{array}\right]=\left[\begin{array}{cc} C_{9} & -C_{10} \end{array}\right]F_{A}\\ \left[\begin{array}{cc} \sigma_{11} & \sigma_{12} \end{array}\right]=\left[\begin{array}{cc} -C_{11} & C_{12} \end{array}\right]F_{B\text{​}}=\left[\begin{array}{cc} -C_{10} & C_{9} \end{array}\right]F_{A}\\ \left[\begin{array}{cc} \sigma_{13} & \sigma_{14} \end{array}\right]=\left[\begin{array}{cc} C_{13} & -C_{14} \end{array}\right]F_{D}’=\left[\begin{array}{cc} C_{9} & -C_{10} \end{array}\right]F_{A}\\ \left[\begin{array}{cc} \sigma_{15} & \sigma_{16} \end{array}\right]=\left[\begin{array}{cc} -C_{15} & C_{16} \end{array}\right]F_{C}’=\left[\begin{array}{cc} -C_{14} & C_{13} \end{array}\right]F_{D}’=\left[\begin{array}{cc} -C_{10} & C_{9} \end{array}\right]F_{A} \end{array}$$

Based on Equation (A5), the output voltage of *U_Z_* is given in Equation (A6):

(A6) $$U_{Z}=\left(\frac{R_{11}+R_{14}}{R_{11}+R_{12}+R_{13}+R_{14}}-\frac{R_{9}+R_{16}}{R_{9}+R_{10}+R_{15}+R_{16}}\right)V_{CC}=\frac{K\times F_{A}\times (C_{9}-C_{10})}{2R+K\times F_{A}\times (C_{9}-C_{10})}V_{CC}$$

In view of *C_9_* ≠ *C_10_*, the value of *U_Z_* is influenced by *M_XY_* and *M_YX_*.

Therefore, the fixed tri-axial dynamometer mentioned in [11,12,13,14] is not able to counteract the extra time produced by the milling cutter moving.

### A.2. Influence of M_E_ on Sensor in [15]

The fixed dynamometer is depicted in Figure A4.

#### A.2.1. *M_XY_* and *M_YX_* Applied on the Sensor

With *M_XY_* and *M_YX_* imposed as shown in Figure A5, the tangential output stresses (*σ_9_*–*σ_12_*) at the center point of Areas 9–12 are presented in the following equation:

(A7) $$\{\begin{array}{l} \left[\begin{array}{cc} \sigma_{9} & \sigma_{10} \end{array}\right]=\left[\begin{array}{cc} C_{9} & -C_{10} \end{array}\right]F_{A}\\ \left[\begin{array}{cc} \sigma_{11} & \sigma_{12} \end{array}\right]=\left[\begin{array}{cc} C_{11} & -C_{12} \end{array}\right]F_{B\text{​}}=\left[\begin{array}{cc} C_{9} & -C_{10} \end{array}\right]F_{A} \end{array}$$

The output value of *U_Y_* is derived in Equation (A8):

(A8) $$U_{Y}=\left(\frac{R_{9}}{R_{9}+R_{12}}-\frac{R_{10}}{R_{10}+R_{11}}\right)V_{CC}=\frac{K\times F_{A}\times (C_{9}+C_{10})}{2R+K\times F_{A}\times (C_{9}-C_{10})}V_{CC}$$

According to the above equation, *U_Y_* under *M_XY_* and *M_YX_* is not cancelled by the sensor circuit.

#### A.2.2. *M_XZ_* and *M_ZX_* Applied on the Sensor

When loaded with *M_XZ_* and *M_ZX_* as plotted in Figure A6, the output stresses (*σ_1_*–*σ_8_*) under *F_A_*, *F_B_* and *M_A_*, *M_B_* are given as follows:

(A9) $$\{\begin{array}{l} \left[\begin{array}{c} \sigma_{1}\\ \sigma_{2} \end{array}\right]=\left[\begin{array}{cc} C_{1} & C_{1}’\\ C_{2} & -C_{2}’ \end{array}\right]\left[\begin{array}{c} F_{A}\\ M_{A} \end{array}\right]=\left[\begin{array}{cc} C_{1} & C_{1}’\\ C_{1} & -C_{1}’ \end{array}\right]\left[\begin{array}{c} F_{A}\\ M_{A} \end{array}\right]\\ \left[\begin{array}{c} \sigma_{3}\\ \sigma_{4} \end{array}\right]=\left[\begin{array}{cc} -C_{3} & C_{3}’\\ -C_{4} & -C_{4}’ \end{array}\right]\left[\begin{array}{c} F_{B}\\ M_{B} \end{array}\right]=\left[\begin{array}{cc} -C_{1} & C_{1}’\\ -C_{1} & -C_{1}’ \end{array}\right]\left[\begin{array}{c} F_{A}\\ M_{A} \end{array}\right]\\ {}[\begin{array}{cc} \sigma_{5} & \sigma_{6} \end{array}]=\left[\begin{array}{cc} -C_{5} & C_{6} \end{array}\right]F_{A}\\ {}[\begin{array}{cc} \sigma_{7} & \sigma_{8} \end{array}]=\left[\begin{array}{cc} C_{7} & -C_{8} \end{array}\right]F_{B}=\left[\begin{array}{cc} C_{5} & -C_{6} \end{array}\right]F_{A} \end{array}$$

where the flexible coefficient symbols *C* and *C’* have the same meanings as mentioned in Section A.1.1.

Based on Equation (A9), the output signals of *U_X_* and *U_Z_* are given below:

(A10) $$U_{X}=\left(\frac{R_{6}}{R_{6}+R_{8}}-\frac{R_{5}}{R_{5}+R_{7}}\right)V_{CC}=\frac{K\times F_{A}\times \left(C_{5}+C_{6}\right)}{2R}V_{CC}$$

(A11) $$U_{Z}=\left(\frac{R_{1}}{R_{1}+R_{1}}-\frac{R_{4}}{R_{3}+R_{4}}\right)V_{CC}=\left(\frac{R+K\times \left(F_{A}\times C_{1}+M_{A}C_{1}’\right)}{2R+2K\times F_{A}\times C_{1}}-\frac{R-K\times \left(F_{A}\times C_{1}+M_{A}C_{1}’\right)}{2R-2K\times F_{A}\times C_{1}}\right)V_{CC}$$

According to Equation (A11), the sensor output of *U_X_* and *U_Z_* varies with *M_XZ_* and *M_ZX_*.

#### A.2.3. *M_YZ_* and *M_ZY_* Applied on the Sensor

As shown in Figure A7, with *M_YZ_* and *M_ZY_* applied on the sensor, the output stresses (*σ_5_*–*σ_8_*) under *M_A_* and *M_B_* are formulated in Equation (A12):

(A12) $$\{\begin{array}{l} \left[\begin{array}{cc} \sigma_{5} & \sigma_{6} \end{array}\right]=\left[\begin{array}{cc} -C_{5}’ & C_{6}’ \end{array}\right]M_{A}\\ \left[\begin{array}{cc} \sigma_{7} & \sigma_{8} \end{array}\right]=\left[\begin{array}{cc} C_{7}’ & -C_{8}’ \end{array}\right]M_{B\text{​}}=\left[\begin{array}{cc} C_{5}’ & -C_{6}’ \end{array}\right]M_{A} \end{array}$$

The output value of *U_X_* is expressed below:

(A13) $$U_{X}=\left(\frac{R_{6}}{R_{6}+R_{8}}-\frac{R_{5}}{R_{5}+R_{7}}\right)V_{CC}=\frac{K\times M_{A}\times \left(C_{5}’+C_{6}’\right)}{2R}V_{CC}$$

It is apparent that *U_X_* is influenced by *M_YZ_* and *M_ZY_* due to the upper plate tilt.

Considering Equations (A8), (A10), (A11), and (A13), the developed sensor reported in [15] is not very suitable for measurement of a movable milling force without any accessory device for position compensation.
